# Addressing microwave irradiation for the facile preparation of nanocrystalline cellulose^1^^✰✰^^★^

**DOI:** 10.1016/j.mex.2026.103912

**Published:** 2026-04-14

**Authors:** K.Y. Lim, K.Y. Foo

**Affiliations:** River Engineering and Urban Drainage Research Centre (REDAC), Universiti Sains Malaysia, Engineering Campus, Seri Ampangan, 14300 Nibong Tebal, Penang, Malaysia

**Keywords:** Alkalization, Bleaching, Hydrolysis, Lignocellulose, Microwave, Nanocrystalline cellulose

## Abstract

This work presents a microwave-assisted technique for the preparation of nanocrystalline cellulose (NCC). The method involves a sequence of controlled chemical treatments, comprises of alkaline delignification (2 % w/v NaOH), chlorine-free bleaching (2 wt% H₂O₂) and an acid hydrolysis step (50 wt% H₂SO₄). Each treatment is precisely outlined, with a clear focus on the underlying objectives, procedure, key observations and the rationale for specific conditions, to ensure reproducibility of the experimental design. The technique is validated through comprehensive characterization, including scanning electron microscopy (nano-whisker shape), transmission electron microscopy (average length = 264.35 nm, diameter = 25.26 nm and aspect ratio = 10.47), X-ray diffraction (*CI* = 65.90 %), fourier-transform infrared spectroscopy, thermogravimetric analysis (T_max_ = 300.71 °C), zeta potential measurement (δ = -44.50 mV) and production yield (61.50 %). The intensification of dipolar rotation and ionic conduction during microwave-assisted processing preserves the crystallinity and stability of the final product, and enables scalable NCC production for diverse applications.

• Facile preparation technique of nanocrystalline cellulose.
• Microwave-assisted alkalization, bleaching and acid hydrolysis reactions.
• Preservation of morphological structure, shape, size, crystallinity, functionality, thermostability and surface charge.

## Graphical abstract

**Figure:**
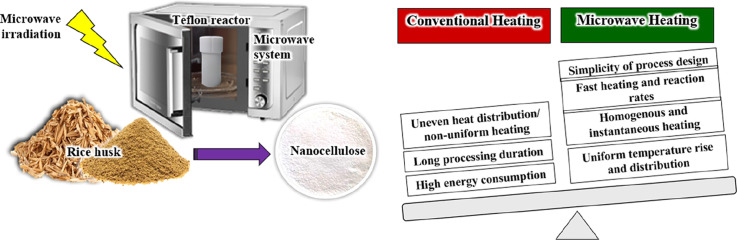

## Specifications table

**Table:** 

**Subject area**	Materials Science
**More specific subject area**	Microwave synthesis technique, Nanomaterial and Resource recovery
**Name of your method**	Microwave synthesis technique of nanocrystalline cellulose
**Name and reference of original method**	1. K.Y. Foo, B.H. Hameed, Recent developments in the preparation and regeneration of activated carbons by microwaves, Advances in Colloid and Interface Science 149 (2009) 19–27. 2. K.Y. Lim, K.Y. Foo, Facile synthesis of nanocrystalline cellulose from rice husk by microwave heating: Evaluation of morphological architectures from the macro-to-nano dimensions, Cellulose 31 (2024) 9661–9679.
**Resource availability**	-

## Background

Nanocellulose, as a new fascinating cellulose-based bio-materials in the scientific community, has been highlighted for its unique supramolecular architecture and exceptional physicochemical attributes. These include nanoscale dimensions, outstanding mechanical and tensile strength, tunable surface chemistry, high thermal stability and crystallinity, intrinsic biodegradability and low cytotoxicity [1,2]. The degradation of amorphous non-cellulosic components from lignocellulosic biomass, and the subsequent transformation into nanoscale cellulose is a multi-step process, which proceeds through a series of sequential operations beginning with alkaline treatment and bleaching, and culminating in controlled acid hydrolysis [3]. Alkaline treatment serves as a critical initial step to disrupt the recalcitrant lignocellulosic matrix. It selectively removes surface impurities, extractives, waxes, lignin and a substantial fraction of hemicellulose, to expedite the access of cellulose microstructure [4]. Bleaching reaction, after alkaline treatment/ pulping, is applied to produce high-purity cellulose with reduced chromophoric content, and to cleave residual glycosidic and ether linkages that tether hemicellulose and lignin to the cellulose surface. This step enhances fiber swelling, flexibility and overall reactivity [5]. Acid hydrolysis is subsequently performed as the defining stage for nanocellulose isolation. Under tightly controlled reaction conditions, the acid selectively cleaves glycosidic linkages and disrupts hydrogen-bonded amorphous regions, liberating nano-crystalline cellulose particles with narrow size distributions [6].

Today, most reported nanocellulose production pathways rely on conventional heating methods, in which thermal energy is transferred to the reaction medium through conduction, convection and radiation. However, this externally driven, surface-dominated heating mechanism often requires longer reaction times to attain thermal equilibrium within the bulk matrix, and may exacerbate temperature gradients, particularly in samples with heterogeneous dimensions or irregular geometries. Such non-uniform heat flux distribution increases the risk of localized overheating and charring, leading to irreversible degradation of the cellulose polymeric backbone and disruption of crystalline domains. Consequently, production yield, structural uniformity and physicochemical consistency of nanocellulose are compromised, thereby limiting the scalability and commercial viability [7]. In response, increasing attention has shifted towards more energy-efficient synthesis techniques. Among all, microwave heating, which enables volumetric dielectric heating at the molecular scale, has emerged as a viable alternative energy source for nanocellulose preparation. Under microwave irradiation, electromagnetic energy is converted into heat through direct interaction between the incident radiation and the material matrix via dipolar polarization (re-orientation of polar molecules under an oscillating electric field) and ionic conduction (electrophoretic migration of dissolved ionic species under the applied electromagnetic field) mechanisms [8]. Given the intrinsic characteristics of these mechanisms, microwave dielectric heating has become a highly valuable tool for nanocellulose processing, owing to its (a) simplicity of process design, (b) rapid, volumetric and spatially homogeneous heating profiles, (c) enhanced energy transfer efficiency, accelerated reaction kinetics and improved reaction selectivity, (d) low thermal inertia and reduced dependence on auxiliary heating media, and (e) significant energy and time savings [9]. These attributes allow microwave-induced reactions to proceed more uniformly and rapidly compared with conventional heating systems [10,11]. Despite these advantages, the complete integration of microwave heating across all key processing stages-alkalization, bleaching and acid hydrolysis remains insufficiently investigated.

## Method details

### Microwave heating

A microwave reactor system [inner dimension: 36 cm (L) x 33 cm (W) x 24 cm (H)], operating at the maximum power and frequency of 1000 W and 2.45 GHz, respectively, was adopted as the potential heat source. The system was equipped with a digital-timer for the control of irradiation duration, and a proportional-integral derivative (PID) controller for the precise regulation of process temperature. A high-temperature Type-K thermocouple was interfaced with the reaction vessel to enable continuous temperature monitoring during the pyrolysis process. Microwave heating was carried out in a Teflon-lined reactor, which was positioned on the rotary platform of the microwave cavity.

### Model cellulosic precursor and biomass pretreatment

Considering the substantial volume of waste generated with limited practical applications, rice husk (*Oryza sativa*), which contains a cellulose fraction of 35–40 %, was selected as a model candidate for nanocellulose preparation. Its abundant availability and cost-effectiveness, make it an attractive resource. The rice husk (RH) samples were meticulously cleaned, dried, and subjected to milling and sieving processes to achieve a particle size with a geometric mean of 1–2 mm.

Biomass pretreatment is a preparatory stage that effectively swells and softens the compact/ rigid structure of lignocellulosic matrix, solubilizes surface impurities/ non-cellulosic components, and enhances the reactivity of cellulose, facilitating the subsequent processing steps. In the study, the biomass pretreatment was conducted by immerging the dried biomass (1–2 mm) in double deionized (DI) water, at a solid-to-liquid ratio of 1 g: 20 mL. The resulting slurry was then transferred to a Teflon-lined pressure reactor, and subjected to microwave-assisted dielectric heating at 100 °C for 1 min. After the reaction, the reactor was cooled to ambient temperature, and the insoluble fraction was collected. The solid residue was washed repeatedly with hot DI water until the supernatant turned visually clear. The clarified solid fraction, obtained through sequential filtration and centrifugation (6000 rpm), was applied for the subsequent alkaline delignification step.

In the process, DI water serves as a heat-transfer medium, which enables uniform temperature distribution minimizing localized temperature gradients, and avoids the formation of inhibitors, that could otherwise promote excessive cellulose depolymerization. Additionally, the application of microwave irradiation could further accelerate water molecule uptake and diffusion, enhancing the penetration and flow within the biomass structure. Under these conditions, the expansion and structural changes of biomass structure promote the loosening of chemical bonds in hemicellulose, lignin and lignin-carbohydrate complexes, exposing cellulose molecules, thereby improve the structural integrity of the resulting nanocellulose.

### Isolation of nanocrystalline cellulose

**Fig. 1:**
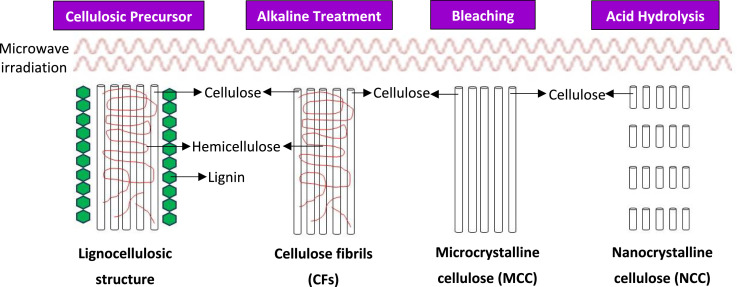
A schematic representation of the nanocellulose extraction protocol.

The isolation of nanocrystalline cellulose could be divided into three principal stages, namely alkalization, bleaching and acid hydrolysis reactions. A schematic representation of the nanocellulose extraction protocol is provided in Fig. 1.

### Alkaline treatment

**1. Main aim:**

Alkaline treatment is a key delignification step that disrupts the recalcitrant lignocellulosic structure, and removes the associated non-cellulosic amorphous fractions (hemicellulose, lignin and other extractives) to facilitate the downstream physicochemical or biochemical processing.

**2. Procedure:**

Alkaline delignification of rice husk pulp was performed using a 2 % (w/v) sodium hydroxide (NaOH) solution. The liquefied pulp was transferred to a Teflon-lined reactor, and NaOH was added at a solid-to-liquid ratio of 1 g: 20 mL. The mixture was subjected to microwave-irradiation at 75 °C for 10 min. The reaction was terminated by quenching with excess cold DI water, and the supernatant containing solubilized lignin was removed by repeated washing until neutral pH (pH 6–7) was achieved. The resulting brownish-black cellulose-rich slurry was collected via sequential centrifugation at 6000 rpm, and dried in a convection oven at 50 °C to a constant mass.

**3. Importance:**

The alkaline delignification process enhances the cellulose purity by selectively removing lignin, hemicellulose and other non-cellulosic constituents that could otherwise impede the quality of nanocellulose. This treatment yields a structurally more uniform and chemically refined cellulose framework, providing an optimal substrate for subsequent crystallization into nanocellulose. The step also improves fiber homogeneity, which supports more efficient downstream bleaching and acid hydrolysis processing.

### Bleaching

**1. Main aim:**

As the second stage of nanocellulose production, bleaching supports the purification of cellulose by depleting residual lignin, hemicellulose and other extractives. The process also degrades phenolic and chromophoric compounds within the cellulosic substrate, which in turn improves both chemical purity and optical quality of the cellulose fractions.

**2. Procedure:**

The bleaching solution was prepared by mixing 2 wt% hydrogen peroxide (H_2_O_2_) solution with 1.0 M sodium hydroxide (NaOH) buffer (stabilizer), with the pH adjusted to 10–11. The alkali-pretreated sample was impregnated in the prepared bleach solution at a solid-to-liquid ratio of 1 g: 20 mL. Bleaching process was carried out in a Teflon-lined reactor under microwave-irradiation at 75 °C for 15 min. The bleaching step was repeated (4 cycles) until the solid sample exhibited a white or off-white color, indicative of microcrystalline cellulose (MCC). Following the bleaching step, the sample was filtered and rinsed sequentially with hot and cold DI water until the wash pH settled at 6–7, to ensure the removal of bleaching solution residues and undesirable side reaction products. The bleached sample was oven-dried at 50 °C until constant weight for the subsequent acid hydrolysis step.

**3. Importance:**

Bleaching is essential to maximize cellulose purity before the critical acid hydrolysis step. The process eliminates residual lignin and other organic impurities that might interfere with the hydrolysis process, or degrade the structural and functional properties of the resulting nanocellulose material.

### Acid hydrolysis

**1. Main aim:**

Acid hydrolysis constitutes a principal method for nanocellulose isolation, in which concentrated acid is applied to selectively break down the amorphous or structurally disordered domains of cellulose, leading to the depolymerization of cellulose into nanoscale crystalline structures under controlled conditions.

**2. Procedure:**

Acid hydrolysis was conducted using 50 wt% sulfuric acid (H_2_SO_4_) as a controlled modification of the conventional 64–65 wt% protocol to minimize excessive depolymerization, over-hydrolysis and sulfation of crystalline domains, while preserving the structural integrity and physicochemical properties of nanocrystalline cellulose. High acid concentrations (64–65 wt%) promote rapid acid-catalyzed cleavage of glycosidic bonds and dehydration reactions, which frequently induce severe cellulose degradation and charring.

In contrast, the 50 wt% H_2_SO_4_ system enables selective removal of amorphous regions, limits secondary degradation and maintains crystalline stability. Pre-bleached cellulose was mixed with 50 wt% H_2_SO_4_ at a solid-to-liquid ratio of 1 g: 20 mL to ensure sufficient acid diffusion into the fibrillar matrix, and controlled disintegration of the cellulose structure. The mixture was heated in a microwave system at 90 °C for 10 min. To quench the hydrolysis reaction, the hydrolyzed material was diluted ten-folds with cold DI water. Centrifugation was performed at 6000 rpm for separation of nanocellulose precipitate and removal of residual acid. The resulting milky-white precipitate was collected, and rinsed repeatedly with excess DI water until neutrality (pH 6–7), with the supernatant replaced by fresh DI water during each washing step. The purified nanocellulose was re-dispersed and homogenized in a water-ice bath under ultrasonication (65 % amplitude) for 30 min to prevent over-heating, particle aggregation and desulfation of the sulfate groups. The sonicated suspension was filtered and oven-dried at 50 °C.

**3. Importance:**

Acid hydrolysis is a critical step to transform bulk cellulose into nanocellulose, specifically in the form of cellulose nanocrystals. By selectively hydrolyzing the less-ordered (amorphous) regions of cellulose, this process markedly enhances the crystallinity, mechanical strength and colloidal stability of the resulting nanomaterial. The distinctive properties conferred through this procedure are pivotal to preserve the structural integrity and functional performance of the final nanocellulose, rendering it suitable for a broad spectrum of advanced applications.

**Fig. 2:**
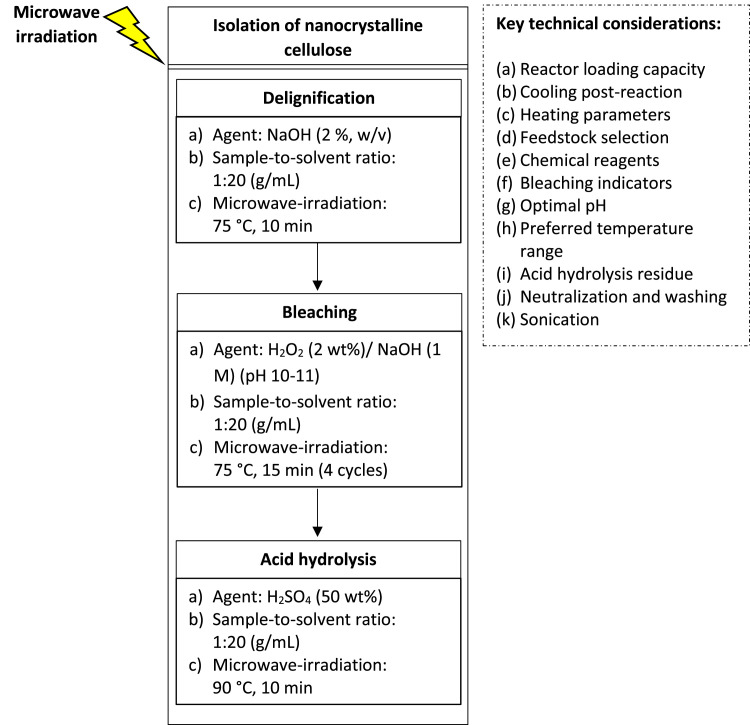
Procedural steps and key technical considerations in the nanocellulose preparation process.

The alterations in the color, physical and chemical features of cellulose products during pretreatment, alkalization, bleaching and acid hydrolysis steps are summarized in Table 1. Fig. 2 presents a flowchart detailing the procedural steps and key technical considerations in the nanocellulose preparation process.

**Table 1:** Overview of the changes in color, physical and chemical features of cellulose products during pretreatment, alkalization, bleaching and acid hydrolysis steps.

Observation	Pretreatment	Alkalization	Bleaching	Acid hydrolysis
**Color**	No appreciable color change	• Formation of a deep black supernatant • Delignified solid transitioned from brownish to light brown	• A pronounced color changes • Initial brown hue progressively lightening to white or off-white	• The nanocellulose suspension displays a milky-white appearance
**Size**	-	Overall particle size remains largely unchanged	The fibers exhibit increased fineness and uniformity	• Smooth and fine texture • Acid hydrolysis substantially reduces particle size • Formation of nanoscale cellulose crystallites
**Physical**	Swollen, moist and water-laden appearance	More fragmented and softer texture	Noticeably reduction in aggregation and clumping	No observable clumps or aggregates
**Chemical**	Does not induce significant chemical transformations, mass loss or structural decomposition	-	-	The nanocellulose forms a translucent, colloidal suspension within the solution

### Technical considerations in the nanocellulose preparation process

(a) **Reactor Loading Capacity:** It is essential to ensure that the volume of the Teflon-lined reactor does not exceed 60 % of its total capacity to allow optimal vapor formation, steam circulation and fluid expansion during the heating process. Exceeding this threshold may hinder heat transfer and compromise reaction efficiency.
(b) **Cooling Post-Reaction:** Rapid cooling or exposure to ambient air immediately after heating could induce thermal shock, potentially damaging the reactor and presenting a risk of burns.
(c) **Heating Parameters:** Key parameters, specifically microwave power, heating temperature, reaction duration and internal pressure must be well-regulated within the specified range to prevent overheating, uneven heating, or under-heating of sample, all of which could adversely affect the quality of the nanocellulose product.
(d) **Feedstock Selection:** The choice of feedstock plays a significant role in nanocellulose synthesis. Biomass with a high cellulose content (≥ 40 %) and low lignin content (≤ 15 %) is preferred, for yield and quality improvement.
(e) **Chemical Reagents:** Rigorous control over the concentration of chemical reagents and solution pH at each extraction stage is essential. This prevents excessive dissolution of cellulosic materials into water-soluble oligomers, which may negatively impact both the yield and fundamental properties of the resulting nanocellulose.
(f) **Bleaching Indicators:** The appearance of brown residues following bleaching reaction is indicative of incomplete removal of lignin, hemicellulose, chromophores and other surface impurities. Such residues may impair the quality of the cellulose, and affect the performance of the subsequent processing steps.
(g) **Optimal pH:** Hydrogen peroxide (H₂O₂) is a weak acid with a dissociation constant (K_d_ = 1.78 × 10⁻¹²) and a pK_a_ of 11.75. Its low dissociation in neutral aqueous solutions requires careful consideration of pH conditions. To ensure the stability of H₂O₂, and avoid its premature decomposition, the pH of the bleaching solution should be regulated within the alkaline range (pH 10–11). A pH exceeding 12 results in an excess of HOO⁻, which can inhibit the formation of hydroxyl radicals (HO^•^), reducing the bleaching efficiency. Furthermore, high alkalinity may induce oxidative damage to the cellulose, leading to a decrease in tensile strength, surface crystallinity and yield.
(h) **Preferred Temperature Range:** The preferred temperature for H₂O₂ bleaching is typically between 60–80 °C, which strikes a balance between reaction efficiency and protection of the cellulose fibers.
(i) **Acid Hydrolysis Residue:** The formation of dark or black discoloration following acid hydrolysis suggests that residual lignin, hemicellulose or other amorphous components were not sufficiently removed during pretreatment, delignification and bleaching steps. These surface impurities are prone to acid-induced degradation and carbonization, resulting in the formation of dark, burnt residues.
(j) **Neutralization and Washing:** Neutralization of reaction mixture to a pH of 6–7 after each extraction step is crucial for the removal of residual impurities, and unreacted materials or chemicals, that may interfere with the subsequent processing stages.
(k) **Sonication:** During sonication, it is necessary to monitor the temperature to prevent excessive heat build-up, which could disrupt the nanocrystal structure. The sonication duration and power should be carefully optimized to avoid over-disintegration of nanocrystals reducing the particle size, crystallinity and thermal stability.

### Quality control

The effectiveness of the microwave-assisted nanocellulose extraction method was justified through an in-depth characterization of the resulting nanocellulose, focusing on the changing morphological features, particle dimensions, crystallographic characteristics, functional attributes, thermal stabilities, surface charge properties and yields.

**Scanning Electron Microscopy (SEM):** The surface morphological characteristics and microstructural properties of the nanocellulose were visualized using SEM (Supra 35VP, Germany) at 10 kV. A diluted (∼0.001 %, w/v) sonicated suspension was deposited onto silver stubs, which were then sputter-coated with gold (Au) to prevent charging during imaging.

**Transmission Electron Microscopy (TEM):** The nano-dimensional profile of the nanocellulose particles were assessed using EF-TEM (Libra 120, Carl Zeiss, Germany) at 120 kV. A 10 μL drop of the sonicated nanocellulose dispersion (∼0.001 %, w/v) was deposited onto a carbon-coated copper (Cu) grid, subsequent by negative-staining using a 2 % ethanolic uranyl acetate solution to enhance contrast. The grid was air-dried before imaging. The particle size distribution, in terms of particle diameter (D), length (L) and aspect ratio (L/D), from the average of 100 runs, was quantified using ImageJ processing software.

**X-ray Diffraction (XRD):** The effects of the microwave-assisted nanocellulose extraction method on the crystalline structure, X-ray diffractogram pattern and crystallinity were evaluated using an X-ray diffractometer (Bruker, Germany) equipped with Cu Kα radiation (λ = 1.5406 Å), with an accelerating voltage of 30 kV and a beam current of 10 mA. The XRD data was collected over a scanning angle range of 2θ = 10–80°, with a step size of 0.03°, and analyzed using the PANalytical X'pert HighScore Plus software (pseudo-Voigt fitting function). The crystallinity index (*CI*) was determined through peak deconvolution and Fourier series analysis, as described by Yao *et al*. [12].

**Fourier Transform Infrared Spectroscopy (FTIR):** The detection of surface functional groups was conducted according to the potassium bromide (KBr) technique using a FTIR-2000 spectrophotometer (PerkinElmer Inc., USA), with spectral data acquired between 4000 and 400 cm⁻¹. A thin transparent film (16 mm) was prepared by mixing, grinding and pressing the dried nanocellulose sample with KBr (1: 100, w/w), followed by manual hydraulic press.

**Thermogravimetric Analysis (TGA):** The thermal stability bahaviour and degradation characteristics of nanocellulose sample were assessed using a thermogravimetric analyzer (TGA 7, PerkinElmer, USA). The dried sample (5–10 mg) was heated from ambient temperature to 600 °C at a ramping rate of 5 °C/min under a continuous nitrogen (N_2_) gas flow of 10 mL/min.

**Zeta potential:** The electrostatic surface charge (attractive or repulsive interactions) of the cellulose nanoparticles was evaluated using a Nano-ZS Zetasizer apparatus (Malvern Instrument, UK). A 0.01 % (w/v) nanocellulose suspension was dispersed in DI water and sonicated for 5 min. The refractive index of each suspension was determined using a digital refractometer (RX-5000 CX, ATAGO, Japan) at room temperature.

**Yield measurement:** The yield (*Y*) of extracted nanocellulose was determined using a gravimetric method (OHAUS Pioneer Balance, PA214C, OHAUS Corporation, USA). The percentage yield (%) was calculated according to Eq. (1) as the ratio of the oven-dry mass of nanocrystalline cellulose obtained after acid hydrolysis (NCC, M₂) to the initial oven-dry mass of the microcrystalline cellulose (MCC, M₁), expressed as:

(1) $$Y\left(\%\right)=\frac{M_{2}}{M_{1}}\times 100$$

### Method validation

The preparation technique was validated in viewpoints of the morphological aspect, shape, particle size, crystallinity, surface chemistry/ functionality, thermal stability, surface charge and yield, as depicted in Table 2 [13]. The detected whisker-shaped nanofibril morphology, with an average diameter of 25.26 nm, a length of 264.35 nm, an aspect ratio of 10.47 and a crystallinity index of 65.90 %, provides quality assurance of the synthesized crystalline nanocellulose. The corresponding composition analysis ascsertained the progressive removal of amorphous and non-cellulosic substances (hemicelluloses, lignin, wax and silica), leading to the enhanced thermal (T_max_ = 300.71 °C) and colloidal (ζ = −44.50 mV) stabilities. A comparison between the reaction duration, production yield and thermal stability across different nanocellulose preparation techniques are tabulated in Table 3. Comparison with the literature findings (Table 3) reinforces the distinct role microwave irradiation, driven by the dipolar polarization and ionic conduction mechanisms [14,15].

**Table 2:** Method validation in viewpoints of the morphological aspect, shape, particle size, crystallinity, surface chemistry/ functionality, thermal stability and surface charge.

Sample	Features								
	Shape	Mean diameter (nm)	Mean length (nm)	Aspect ratio	Yield (%)	CI (%)	Surface chemistry	T_max_ ( °C)	Zeta potential (mV)
Nanocellulose	Whisker shape	25.26 ± 1.16	264.35 ± 7.6	10.47	61.50	65.90	Hydroxyl and sulfate functional groups	300.71	−44.50

**Table 3:** Comparison of reaction time, production yield and thermal stability across different nanocellulose preparation techniques.

Precursor	Reaction time			Total reaction time (min)	Preparation technique	Yield (%)	Thermal stability (T_max_, °C)	Reference
	Alkaline treatment (min)	Bleaching (min)	Hydrolysis (min)					
Rice husk	10	60	10	80	M-M-M	61.50	300.71	This study
*Gelidiella aceroso* red seaweed	30	240	30	300	M-C-C	30.00	-	[20]
Chestnut shell	60	60	59	179	C-C-M	34.10	-	[21]
Cotton	120	120	120	360	C-C-M	58.00	-	[22]
Jute fiber	120	90	30	240	C-C-C	38.00	-	[23]
Sugarcane trash	720	480	90	1290	C-C-C	-	361.53	[24]
Rice husk	480	420	90	990	C-C-C	33.78	351.00	[25]

C: Conventional heating; M: Microwave heating.

In microwave systems, thermal energy arises from direct interaction between electromagnetic radiation and molecular dipoles within the bulk material, rather than from external surface heat transfer [16]. The oscillation of polar molecules, migration of ionic species and rapid dipole rotation generate internal heat, leading to the formation of a temperature gradient that decreases from the interior towards the surface, opposite to that observed in conventional heating systems, where heat propagates from the exterior to the interior [17]. This internally generated thermal gradient enhances heat transfer efficiency, and increases the reactivity of the cellulose matrix and chemical reagents. Such conditions facilitate deeper penetration of chemical agents, and promote effective removal of non-cellulosic constituents, amorphous domains and susceptible chemical linkages during alkalization, bleaching and acid hydrolysis. Consequently, the internal regions of raw rice husk, cellulose fibers (CFs) and microcrystalline cellulose (MCC) experience more uniform and efficient heating under microwave irradiation, even at lower bulk temperatures and shorter processing durations. The reduced processing time observed in this study highlights the energy-efficiency and mechanistic effectiveness of microwave heating. This approach preserves the fundamental structural characteristics of NCCs while overcoming the intrinsic limitations of conventional surface-heating systems.

NaOH exhibits strong microwave absorption during dielectric heating. According to Foo and Hameed [18], localized heating generated by dipole rotation and intermolecular friction under microwave irradiation supplies the energy required for NaOH molecules to align with the applied electric field. This interaction stimulates (a) the nucleation and collapse of microbubbles within the lignocellulosic polymer matrix, (b) accelerates alkali diffusion into the interfibrillar regions, and (c) improves mass transfer, which facilitates rapid detachment, loosening and swelling of the cementing lignocellulosic network [14]. Consequently, cellular constituents are progressively released from the structural matrix.

Hydrogen peroxide (H₂O₂), a strong oxidizing agent, oxidizes susceptible organic constituents to H₂O and CO₂. In pulp bleaching system, it contributes to long-term stabilization and protection against damage induced by reactive oxygen species (ROS). In environmental engineering applications, microwave irradiation in closed-vessel digestion system, coupled with H₂O₂ has recently been explored as a microwave-assisted oxidation approach. This process enhances the decomposition rate of hydrogen peroxide into hydroxyl radicals (•OH) through dielectric polarization under microwave energy. During the reaction, cationic counterions may interact with lignin side chains and cleave alkyl-aryl ether bonds within lignin-carbohydrate complexes via electrophilic substitution [13]. This mechanism supports progressive lignin removal from chemically purified rice husk cellulose fibres (RH-CFs).

Dipolar polarization represents the principal mechanism governing microwave-assisted acid hydrolysis. Oscillation of electric field induces rapid molecular rotation, and provides simultaneous heating and micro-scale mixing. Sulfuric acid (H₂SO₄) absorbs microwave energy, and generates localized hotspots that produce shock-wave effects at the surface of rice husk microcrystalline cellulose (RH-MCC). These effects promote preferential erosion of amorphous regions along the axial direction of the cellulose structure, and lead to the formation of nanocellulose fragments. Within this framework, the synergistic interaction between microwave irradiation and H₂SO₄ treatment disrupts strong hydrogen bonding within the cellulose matrix through rotation of polar hydroxyl groups [19]. This leads to the progressive defibrillation of chemically purified RH-MCC into rice husk nanocellulose (RH-NCCs), concomitant with the partial esterification of cellulose nanocrystallites.

## Limitations

(a) **Variability in Raw Material Quality:** The composition of rice husk can exhibit significant variability according to geographical location, cultivation techniques and harvesting conditions. Such variability may lead to fluctuations in cellulose content (between 35 % to 40 %), which in turn affects both yield and purity of the extracted nanocellulose.
(b) **Risk of Cellulose Degradation:** Excessive microwave irradiation or improper temperature regulation may engender degradation of cellulose, particularly when the heating duration is not meticulously controlled. Prolonged exposure to elevated microwave power could result in undesirable chemical alterations or thermal decomposition of cellulose.
(c) **Potential for Cellulose Hydrolysis and Substitution:** While alkaline treatment effectively removes non-cellulosic components, it could trigger hydrolysis of cellulose if prolonged exposure occurs. This process can lead to the depolymerization of cellulose, reducing its molecular weight, and compromising its structural and functional properties.
(d) **Process Repetitiveness:** Achieving a high degree of whiteness or high-quality cellulose often necessitates multiple cycles of bleaching. This repetitive process may result in extended processing durations and higher chemical usage, diminishing the preparation efficiency.
(e) **Environmental Considerations:** The use of hydrogen peroxide, concentrated sulfuric acid and other bleaching agents raises concerns about the environmental impact of their handling, storage and disposal.

## Ethics statements

No ethic statements to declare.

## CRediT author statement

K.Y. Lim: Writing – Original draft, Visualization, Methodology, Formal analysis, Data curation. K.Y. Foo: Writing – Review & editing, Validation, Supervision, Resources, Project administration, Funding acquisition, Conceptualization.

## Declaration of interests

The authors declare that they have no known competing financial interests or personal relationships that could have appeared to influence the work reported in this paper.

## Data Availability

Data will be made available on request.
